# Sociodemographic differences in low back pain: which subgroups of workers are most vulnerable?

**DOI:** 10.1186/s12891-024-07970-5

**Published:** 2024-10-26

**Authors:** Johannes Beller, Stefanie Sperlich, Jelena Epping, Batoul Safieddine, Janice Hegewald, Juliane Tetzlaff

**Affiliations:** 1 Hannover Medical School, Hannover, Germany; 2 Federal Institute for Occupational Safety and Health (BAuA), Berlin, Germany; 3 Center for Public Health and Health Care, Medical Sociology Unit, Hannover Medical School, Carl- Neuberg-Str. 1, 30625 Hannover, Germany

**Keywords:** Back pain, Social inequality, Socioeconomic status, SES, Vulnerable groups

## Abstract

**Background:**

Low back pain (LBP) is a common health problem in workers that contributes to work disability and reduces quality of life. However, studies examining vulnerable groups in relation to sociodemographic differences in LBP remain scarce. Therefore, the current study investigates which sociodemographic groups of workers are most affected by LBP.

**Methods:**

Data from the 2018 BIBB/BAuA employment survey were used (*N* = 16252). Age, education, occupational group, income, working hours, atypical working time, relationship status, and having children were used as sociodemographic predictors. Gender-stratified logistic regression analyses and intersectional classification tree analyses were conducted.

**Results:**

A higher prevalence of LBP was observed for women compared to men. Significant differences in LBP emerged for age, working hours, atypical working time, occupational group and education, with some gender differences in the importance of predictors: Age was a significant predictor mostly in men as compared to women, atypical working hours had a slightly greater effect in women, whereas differences in LBP according to the occupational group were more pronounced for men. Vulnerable groups were found to be women who work in occupations other than professionals or managers, work atypical hours and have an intermediate or low educational level as well as men who work as skilled agricultural workers, craft workers, machine operators, or elementary occupations and are between 35 and 64 years old.

**Conclusions:**

Thus, workers with certain occupations and lower levels of education, middle-aged men and women with unfavourable working time characteristics are most affected by low back pain. These groups should be focused on to potentially increase healthy working life and prevent work disability.

## Background

Low back pain (LBP) is a major public health problem and the leading cause of disability worldwide [1–3], with a global 12-month prevalence of about 37% among adults [2, 4]. LBP can lead to severe consequences, including activity limitations that have been increasing among working-age adults in the US and most of Europe [5–9]. Furthermore, LBP is a significant predictor of psychological problems, with individuals experiencing approximately double the risk of depression compared to those without LBP [10, 11]. Additionally, in occupational settings, LBP results in tremendous costs due to reduced work ability and is one of the most common causes of work absence and disability [2, 12–16]. Even in comparatively young and highly educated samples, LBP has been reported to be a common and potentially debilitating health complaint [17].

Sociodemographic differences in morbidity were reported by a large body of research with higher prevalence typically occurring in more socially disadvantaged groups [18, 19]. With respect to LBP, those working in manual occupations exhibit much higher prevalences of LBP as compared to non-manual workers [20–23]. Additionally, lower educational attainment as well as having a lower income are also associated with a greater likelihood of having LBP [24, 25]. For example, in a study conducted among older Japanese, Ikeda and colleagues found clear differences in LBP prevalence by educational attainment level [26]. Many studies have also shown that atypical working hours are associated with increased LBP risks [27, 28]. Further disparities are evident across the lifespan and gender, with higher ages and women typically reporting higher a prevalence and a different presentation of LBP [2, 29–32].

While previous studies have investigated socioeconomic differences in low back pain (LBP) prevalence, they have often relied on composite scores or single measures such as income, providing an incomplete picture [33]. To address this gap, the current study aims to examine multiple sociodemographic indicators and their interactions simultaneously, including education, income, occupation, age, gender, and working time. Commonly used logistic regression analyses as well as modern underutilized tree-based intersectional analyses are conducted on data from a large population-based employment survey. Additionally, also going beyond most previous studies, concrete occupational groups spanning the whole occupational spectrum are differentiated. Thereby, the combined effects of socioeconomic characteristics on LBP prevalence can be better understood and the most vulnerable subgroups of workers can be identified.

## Methods

### Sample

Data were used from the 2018 BIBB/BAuA Employment Survey, which is a cross-sectional study that collects information about the German workforce [34]. To be included in the survey, participants had to meet the following criteria: Participants are 15 years of age or older, must work for 10 h per week or more, and have an adequate command of the German language. The sampling procedure involved a random-digital-dialing approach for both landline and mobile numbers. The interviews lasted about 40 min on average and focused on sociodemographic variables, work activities, working conditions and health. Participants provided informed consent and all procedures were in accordance with German law and the 1964 Helsinki declaration and its later amendments. The BAuA ethics committee approved the survey (EK007_2017 January 9, 2017). After omitting participants with missing values listwise, a final sample with *N* = 16,252 participants resulted.

### Measures

Low back pain (LBP) was assessed via the following question: “Please tell me if you have had any of the following health problems during work or on working days in the past 12 months. We are interested in complaints that occurred frequently: Low back pain”. Participants could choose to respond with “yes” or “no”. As predictors age, gender, presence of children, marital status, working hours, atypical working time, occupation (classified into 9 groups), income, and education level (categorized as high, intermediate, or low) were included, as described more fully in Appendix Table A1.

### Data analysis

First, descriptive statistics of all variables were calculated and presented as frequencies and percentages for categorical variables, and as means and standard deviations for continuous variables. Additionally, when comparing groups, standardized (mean) differences (SMDs) were calculated. SMD is a measure of distance between two groups [35, 36]. It represents a measure of effect size that expresses the difference in central tendency (mean difference in the case of continuous variables and prevalence difference in the case of dichotomous variables) between two groups in terms of the pooled standard deviation. As it is standardized, comparison across variables on different scales, including metric and binary variables, is possible.

Then, logistic regression analysis was used to study the additive effects of the predictors on the prevalence of low back pain. The regression analyses were stratified by sex and included all covariates (age-group, having children, partnership status, working hours, atypical hours, occupation, income and education). The results were reported as odds ratios (OR) with 95% confidence intervals (CI).

To investigate possible interactive effects in predicting vulnerable groups in LBP, p-value based classification tree analyses were used [37]. In this approach, the data are recursively partitioned into homogeneous subgroups with similar levels of LBP based on the significance of the association between LBP and the included variables. Utilizing a minimum p-value threshold of *p* < .01 for splits and imposing a maximum tree depth of 3, the analyses were conducted employing the “party” package in R statistical software, which implements unbiased recursive partitioning techniques for classification tree analysis.

## Results

Table 1 shows an overall LBP prevalence of 44.15%, with a higher prevalence in women (47.83%) than men (40.60%). Sociodemographic characteristics differed significantly between men and women, except for relationship status. The largest differences were observed in working hours (men worked more on average; SMD = 0.76) and occupation (SMD = 0.72). Men worked more often especially in managerial, craft, and machine operator roles, while women worked more often in technician, clerical, and service roles. Professional roles were similarly distributed.

**Table 1:** Low back pain, and Sociodemographic Characteristics in Working men and women (*N* = 16252)

	Stratified by Gender					
	Overall	Male	Female	*p*		SMD
N	16,252	8259	7993			
Low back pain = TRUE (%)	44.15	40.60	47.83	< 0.001		0.15
Age-group (%)				< 0.001		0.19
15–24	2.94	3.71	2.15			
25–34	14.78	16.65	12.85			
35–44	20.56	20.90	20.21			
45–54	31.66	29.47	33.92			
55–64	27.96	26.54	29.43			
65+	2.10	2.74	1.45			
Children = has children (%)	32.78	31.52	34.08	0.001		0.06
Relationship = in relationship (%)	55.32	55.13	55.52	0.623		0.01
Working hours (mean (SD))	38.25 (11.59)	42.29 (10.13)	34.07 (11.52)	< 0.001		0.76
Atypical Hours = Yes (%)	45.07	48.75	41.27	< 0.001		0.15
Occupation (%)				< 0.001		0.72
1 Managers	6.60	8.43	4.72			
2 Professionals	26.85	27.79	25.87			
3 Technicians	28.03	22.96	33.27			
4 Clerical Support Workers	10.39	6.84	14.05			
5 Service Workers	10.44	6.94	14.06			
6 Skilled Agricultural workers	1.10	1.72	0.46			
7 Craft workers	8.10	14.17	1.83			
8 Machine Operators	4.95	8.25	1.55			
9 Elementary Occupations	3.54	2.92	4.19			
Income (mean (SD))	3.54 (3.55)	4.25 (3.97)	2.81 (2.87)	< 0.001		0.42
Education (%)				< 0.001		0.20
High	53.62	53.30	53.95			
Intermediate	31.95	29.05	34.96			
Low	14.43	17.65	11.10			

Notes. p = p-value based on t-test and Chi²-test where appropriate; SMD = Standardized (Mean) Difference

Table 2 shows the differences in sociodemographic characteristics of women by LBP status. Women who reported LBP differed descriptively from those who did not in all variables, with occupation (SMD = 0.33), education (SMD = 0.32), and atypical hours (SMD = 0.17) exhibiting the largest standardized differences. Men who reported LBP differed descriptively from those who did not in all variables, with occupation (SMD = 0.37), education (SMD = 0.35), and atypical hours (SMD = 0.14) exhibiting the largest standardized differences (Table 3).

**Table 2:** Differences in Sociodemographic Characteristics of Women by Low back pain (*N* = 7993)

	Stratified by Low Back Pain				
	No LBP	LBP	*p*		SMD
N	4170	3823			
Low back pain = TRUE (%)	0.00	100.00	< 0.001		
Age-group (%)			< 0.001		0.11
15–24	1.85	2.48			
25–34	13.21	12.45			
35–44	21.15	19.17			
45–54	33.65	34.21			
55–64	28.27	30.68			
65+	1.87	0.99			
Children = has children (%)	35.90	32.10	< 0.001		0.08
Relationship = in relationship (%)	56.67	54.28	0.034		0.05
Working hours (mean (SD))	33.67 (11.82)	34.51 (11.17)	0.001		0.07
Atypical Hours = Yes (%)	37.19	45.72	< 0.001		0.17
Occupation (%)			< 0.001		0.33
1 Managers	5.32	4.05			
2 Professionals	31.49	19.75			
3 Technicians	31.25	35.47			
4 Clerical Support Workers	14.36	13.71			
5 Service Workers	11.20	17.19			
6 Skilled Agricultural workers	0.38	0.55			
7 Craft workers	1.70	1.96			
8 Machine Operators	1.44	1.67			
9 Elementary Occupations	2.85	5.65			
Income (mean (SD))	2.93 (3.02)	2.67 (2.69)	< 0.001		0.09
Education (%)			< 0.001		0.32
High	61.18	46.06			
Intermediate	30.67	39.63			
Low	8.15	14.31			

Notes. p = p-value based on t-test and Chi²-test where appropriate; SMD = Standardized (Mean) Difference

**Table 3:** Differences in Sociodemographic Characteristics of Men by Low back pain (*N* = 8259)

	Stratified by Low Back Pain				
	No LBP	LBP	*p*		SMD
N	4906	3353			
Low back pain = TRUE (%)	0.00	100.00	< 0.001		
Age-group (%)			< 0.001		0.16
15–24	4.04	3.22			
25–34	17.59	15.27			
35–44	21.73	19.68			
45–54	28.21	31.32			
55–64	25.03	28.75			
65+	3.40	1.76			
Children = has children (%)	32.04	30.75	0.223		0.03
Relationship = in relationship (%)	54.59	55.92	0.240		0.03
Working hours (mean (SD))	42.01 (10.33)	42.69 (9.82)	0.003		0.07
Atypical Hours = Yes (%)	45.84	53.00	< 0.001		0.14
Occupation (%)			< 0.001		0.37
1 Managers	9.19	7.31			
2 Professionals	32.41	21.03			
3 Technicians	23.58	22.04			
4 Clerical Support Workers	6.81	6.89			
5 Service Workers	6.83	7.10			
6 Skilled Agricultural workers	1.26	2.39			
7 Craft workers	10.93	18.91			
8 Machine Operators	6.85	10.29			
9 Elementary Occupations	2.14	4.06			
Income (mean (SD))	4.48 (4.04)	3.92 (3.84)	< 0.001		0.14
Education (%)			< 0.001		0.35
High	59.66	43.99			
Intermediate	27.13	31.85			
Low	13.21	24.16			

Notes. p = p-value based on t-test and Chi²-test where appropriate; SMD = Standardized (Mean) Difference

Next, logistic regression analysis revealed socioeconomic differences in LBP. As shown in Table 4, women working more hours (OR = 1.01, 95% CI [1.01; 1.02], *p* < .001) and atypical hours (OR = 1.21, 95% CI [1.09; 1.33], *p* < .001) had significantly higher odds of LBP. Additionally, women in technical, clerical, service, and elementary occupations had significantly higher odds of LBP compared to professionals. Those with intermediate (OR = 1.40, 95% CI [1.26; 1.57], *p* < .001) or low education (OR = 1.72, 95% CI [1.45; 2.03], *p* < .001) also had higher odds of LBP. Age was not strongly related to LBP in women, except for the 65 + group, which had significantly lower odds (OR = 0.46, 95% CI [0.28; 0.77], *p* = .003).

**Table 4:** *Logistic regression results Predicting Low Back Pain in women and men*

		Women			Men		
	OR	95%-CI	*p*		OR	95%-CI	*p*
Age-group							
Age-group 15–24 (Ref.)	-	-	-		-	-	-
Age-group 25–34	0.82	[0.58; 1.14]	0.231		1.30	[0.99; 1.69]	0.062
Age-group 35–44	0.80	[0.58; 1.12]	0.195		1.36	[1.04; 1.78]	0.025
Age-group 45–54	0.84	[0.60; 1.15]	0.266		1.58	[1.21; 2.05]	< 0.001
Age-group 55–64	0.85	[0.62; 1.17]	0.323		1.65	[1.26; 2.15]	< 0.001
Age-group 65+	0.46	[0.28; 0.77]	0.003		0.86	[0.58; 1.28]	0.458
Children (Yes)	0.95	[0.84; 1.06]	0.361		0.97	[0.87; 1.09]	0.656
Partnership (Yes)	0.98	[0.89; 1.08]	0.759		1.07	[0.97; 1.20]	0.171
Working Hours	1.01	[1.01; 1.02]	< 0.001		1.01	[1.00; 1.01]	0.008
Atypical Hours (Yes)	1.21	[1.09; 1.33]	< 0.001		1.16	[1.06; 1.28]	0.002
Occupation							
2 Professionals (Ref.)	-	-	-		-	-	-
1 Managers	1.07	[0.86; 1.35]	0.527		1.08	[0.91; 1.31]	0.367
3 Technicians	1.58	[1.39; 1.80]	< 0.001		1.23	[1.07; 1.41]	0.003
4 Clerical Support Workers	1.35	[1.14; 1.58]	< 0.001		1.28	[1.05; 1.56]	0.016
5 Service Workers	1.79	[1.51; 2.13]	< 0.001		1.25	[1.01; 1.53]	0.039
6 Skilled Agricultural workers	1.48	[0.76; 2.89]	0.249		1.95	[1.37; 2.79]	< 0.001
7 Craft workers	1.34	[0.94; 1.89]	0.107		1.95	[1.65; 2.32]	< 0.001
8 Machine Operators	1.21	[0.83; 1.77]	0.312		1.51	[1.24; 1.85]	< 0.001
9 Elementary Occupations	2.34	[1.80; 3.07]	< 0.001		2.12	[1.58; 2.83]	< 0.001
Income	0.99	[0.97; 1.01]	0.250		0.98	[0.97; 1.00]	0.010
Education							
High (Ref.)	-	-	-		-	-	-
Middle	1.40	[1.26; 1.57]	< 0.001		1.23	[1.10; 1.39]	< 0.001
Low	1.72	[1.45; 2.03]	< 0.001		1.68	[1.45; 1.94]	< 0.001

Notes. OR = Odds Ratio; 95%-CI = 95% Confidence Interval; p = p-value

For men, higher age was associated with increased odds of LBP, especially for ages 35–64. Additionally, men working more hours (OR = 1.01, 95% CI [1.00; 1.01], *p* = .008) or atypical hours (OR = 1.16, 95% CI [1.06; 1.28], *p* = .002) had higher LBP odds. Those in technical, clerical, service, agricultural, craft, machine operation, and elementary occupations showed higher LBP odds compared to professionals. Men with intermediate (OR = 1.23, 95% CI [1.10; 1.39], *p* < .001) or lower education (OR = 1.68, 95% CI [1.45; 1.94], *p* < .001) also had higher LBP odds than those with higher education.

**Fig. 1:**
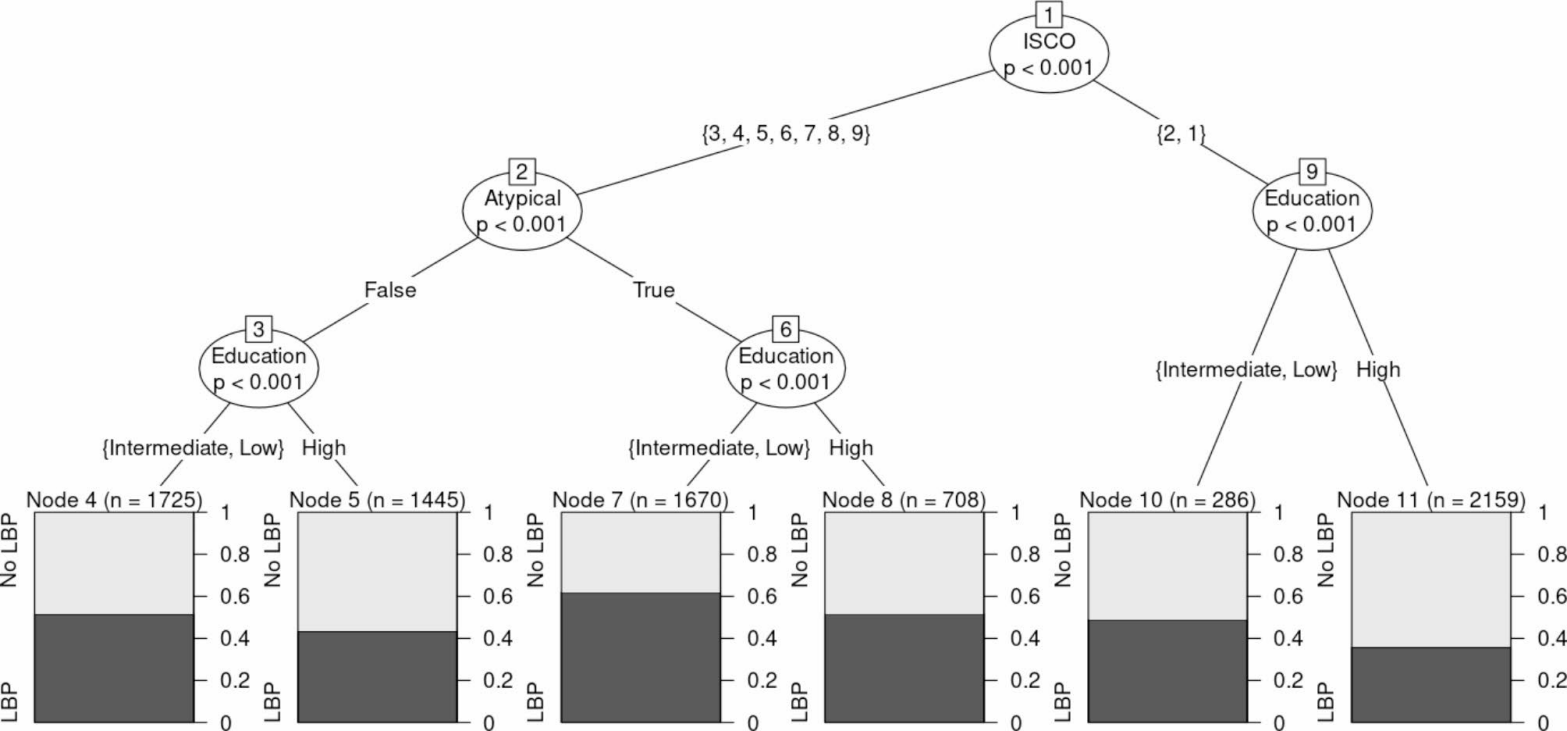
*Classification Tree Analysis Predicting Low Back Pain in Women*

Finally, gender-stratified recursive partitioning analysis was conducted to identify most vulnerable subgroups. As depicted in Fig. 1, the resulting classification tree has six terminal nodes, each representing a subgroup of women with significantly different prevalences of LBP. Occupation emerged as the most important predictor. The most vulnerable group of women are those who work in occupations other than professionals or managers (ISCO groups 3 to 9), have atypical work hours and up to an intermediate level of education. This group has a prevalence of LBP of 61.98%, which is much higher than the overall prevalence of 47.83%. The least vulnerable group of women are those who work as professionals or managers (ISCO groups 1 or 2) and have a higher educational level. This group has a prevalence of LBP of 35.66%, which is much lower than the overall prevalence. Thus, as compared to the least-vulnerable group, prevalence of LBP was much higher in the most vulnerable group. Here again, age had no significant association with LBP (Fig. 1).

**Fig. 2:**
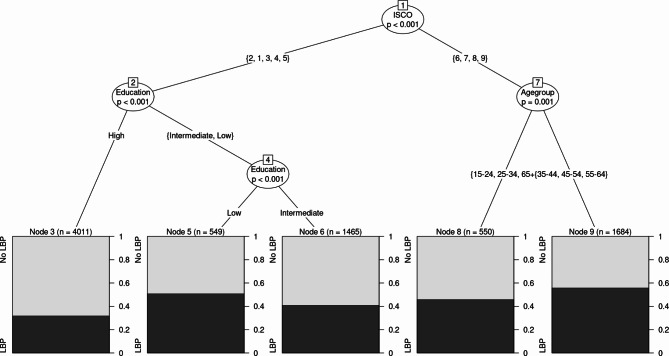
*Classification Tree Analysis Predicting Low Back Pain in Men*

As depicted in Fig. 2, the resulting classification tree for men has five terminal nodes, each representing a subgroup of men with significantly different prevalences of LBP. Occupation again emerged as the most important predictor. The most vulnerable group of men are those who work as skilled agricultural workers, craft workers, machine operators, or elementary occupations (ISCO groups 6 to 9) and are between 35 and 64 years old. This group has a prevalence of LBP of 55.94%, which is much higher than the overall prevalence of 40.60%. The least vulnerable group of men are those who work as professionals, managers, technicians, clerical support workers, or service workers and have a higher educational level. This group has a prevalence of LBP of 31.84%, which is much lower than the overall prevalence. Thus, as compared to the least-vulnerable group, prevalence of LBP was nearly doubled in the most vulnerable group.

## Discussion

Examining sociodemographic differences in low back pain (LBP) among a population-based sample of German workers, it was found that LBP was most strongly associated with high working hours, atypical working time, low-skilled white- and blue-collar occupational groups, and lower educational levels, but not with income, relationship status, or having children. Importantly, the strengths of these associations varied between men and women: For men, age and occupation had stronger effects, whereas atypical working hours were more important in women. Furthermore, vulnerable groups could be identified that had particularly high odds of LBP in the population, such as middle-aged men who worked in low-skilled blue-collar occupations.

These results confirm and extend previous studies. In accordance with prior research, age, education, occupation, and working hours were found to be linked to LBP, and gender differences were noted [2, 20, 38]. Going beyond most previous studies, this study examined distinct occupational groups, allowing more specific identification of vulnerable populations, such as those working in elementary occupations. Typically, previous research had not specially focused on occupational differences or were focused on ultra-specific occupational groups [16, 17, 23]. For example, besides those working in elementary occupations, women working as service workers and men working as craft workers were found to have especially high odds of reporting LBP even when controlling for age, education and other covariates. Evidence for intersectional effects among sociodemographic characteristics were also found, particularly age, education, and occupation in men, and occupation, education, and atypical working time in women, underscoring the importance of this perspective in public health [39]. The findings are also broadly consistent with international data on LBP prevalence and risk factors, while also revealing some unique patterns. For example, a study in the United States by Shmagel and colleagues [40] also found that LBP was more prevalent among older adults and those with lower education levels. However, this study also revealed a more complex relationship with age, especially among women, where the association was not as strong. Research from other cultural contexts provides further context for these findings. In Japan, Ikeda and colleagues reported socioeconomic inequalities in LBP among older adults, with for example lower education being associated with higher LBP prevalence [26]. However, socioeconomic differences appeared much larger in the current study, even when controlling for occupation, implying that socioeconomic factors may play a crucial but differential role in LBP prevalence across different cultural and ethnic groups.

Of theoretical importance, the current results support the intersectional perspective on the occurrence of low back pain. The intersectional perspective posits that multiple socioeconomic categories (such as gender, age, socioeconomic status, and occupation) interact to create unique experiences of health and illness [41, 42]. This approach goes beyond most previous studies on LBP, which typically focus on single risk factors or additive effects, by examining the complex interplay and synergistic effects of multiple social determinants. By adopting an intersectional lens, the current study reveals how combinations of demographic and occupational factors can amplify or mitigate the risk of LBP in ways that may not be apparent when examining these factors in isolation. The practical importance of this study lies in its potential to inform targeted interventions and policies aimed at reducing the burden of LBP in the workplace. LBP is a leading cause of disability worldwide, with substantial economic implications due to lost productivity and healthcare costs. By identifying vulnerable subgroups within the working population, these results can guide the development of more targeted and thus potentially more effective prevention and management strategies, potentially leading to significant improvements in worker health and economic productivity. For example, for men in occupations that involve heavy physical labour, such as skilled agricultural workers, craft workers, machine operators, or elementary occupations, who may have higher rates of LBP due to the nature of their work with increasing age, preventive measures that reduce their workload and improve their work environment at older age may be effective. For women with atypical working hours, such as shift workers, who may have higher levels of LBP due to the disruption of their circadian rhythms and sleep quality, interventions that improve their sleep hygiene and promote their recovery such as improved family-work balance may be beneficial. However, further analyses with even finer occupational groups might be needed to gain insight on more specific occupational risk groups.

The current study has several limitations that should be acknowledged. First, the data were based on self-report, which may introduce biases such as recall errors, social desirability, or health bias. Similarly, information about LBP was based on a single item. This item did not specify the type of LBP. Finer distinctions might have enabled a more complete analysis of sociodemographic differences in LBP, for example whether LBP is caused by muscle tension, which may be introduced by psychosocial stress in many cases, or progressive wear and tear. Therefore, future studies should use more refined measures of LBP that capture its different dimensions and characteristics [43]. Additionally, the study design was cross-sectional, which limits the ability to draw causal inferences and examine temporal relationships between the variables [44]. Moreover, cross-sectional studies cannot capture possible temporal changes in LBP and differences in the associations of sociodemographic variables with LBP over time. Therefore, longitudinal studies are needed. Another limitation of our study is that we did not account for body mass index (BMI), overall physical functioning or other clinical covariates regarding LBP. For example, BMI is known to be a significant factor in the development and experience of low back pain, particularly in occupational settings [45–47]. Future studies should incorporate clinical covariates such as these to provide a more comprehensive understanding of the interplay of risk factors for work-related low back pain across different sociodemographic groups. Regarding the statistical power of the study, with a sample size of *N* = 16,252 participants (*N* = 8259 and *N* = 7993 for men and women) and a low back pain prevalence of 44.2% (40.6% and 47.8% for men and women) the study strongly exceeds the commonly recommended minimum of 10 events per predictor variable for logistic regression analysis, thereby ensuring reliable and stable parameter estimates [48]. This also holds for the large sample sizes within the recursive partitioning analyses [37]. Consequently, the study appears well-powered to detect significant associations and interactions between sociodemographic factors and LBP, supporting the validity and generalizability of the findings.

Thus, despite some limitations, sociodemographic subgroups that are most affected by LBP could be identified, such as individuals with certain occupations and lower levels of education, men of older age and women with unfavourable working time characteristics. These findings have implications for the prevention and management of LBP in the workplace, as well as for the promotion of healthy working life and well-being. Future studies should replicate and expand upon these results, using more objective and comprehensive measures of LBP, and employing longitudinal designs to examine mechanisms and temporal changes [49, 50].

## Conclusions

Sociodemographic differences in low back pain (LBP) among German workers were investigated, with a focus on finding intersectional vulnerable subgroups. Findings revealed that LBP is most consistently associated with high working hours, atypical working time, low-skilled occupational groups, and lower educational levels, with notable gender differences in the strength of these associations. Specific vulnerable groups were uncovered in the intersectional analyses, such as middle-aged men in blue-collar occupations and women in non-professional and non-managerial occupations with atypical hours and lower education. These results underscore the complex nature of LBP and emphasize the need for tailored, gender-specific prevention and management strategies that consider multiple sociodemographic factors. Future research should build upon these findings using longitudinal designs and more refined measures of LBP to further elucidate causal pathways for identifying vulnerable groups.

## Appendix

**Table A1:** Measurement of study variables

Predictor	Measurement
Age	Age was measured in years.
Gender	Gender was measured as male or female.
Children	Participants who indicated that they had any children under the age of 18 living in their household were coded as “yes, has children”, otherwise “no, doesn’t have children”.
Relationship	Participants who indicated that they were currently married were classified as “yes, married”, otherwise as “no, not married”.
Working hours	Working hours were measured as the weekly working hours (in 1-hour units) reported by participants in their main occupation.
Atypical working time	Participants who indicated that they either worked on Saturdays or Sundays at least once a month or indicated that they did not usually work within 7:00–19:00 were classified as “yes, working atypical hours”, otherwise “no, working regular hours”.
Occupation	Participants were classified into nine occupational groups according to the International Standard Classification of Occupations (ISCO): 1 Managers, 2 Professionals, 3 Technicians, 4 Clerical Support Workers, 5 Service Workers, 6 Skilled Agricultural workers, 7 Craft workers, 8 Machine Operators, and 9 Elementary Occupations.
Income	Income was measured as the reported monthly income from the participants’ occupational activity in thousands of Euros.
Education	Participants were categorized into the three educational levels based on their highest educational attainment of “High” (at least a “Hochschulreife”, which is equivalent to a general qualification for university entrance or advanced high school diploma), “Intermediate” (“Mittlere Reife”, akin to an intermediate school leaving certificate or secondary education diploma), and “Low” (up to a “Hauptschulabschluss”, comparable to a basic secondary school leaving certificate).

## Data Availability

The data that support the findings of this study are available from the Federal Institute for Vocational Education and Training (BIBB) at https://www.bibb.de/en/2815.php (doi:10.7803/501.18.1.1.10).
